# Renal manifestations of tuberous sclerosis complex: patients’ and parents’ knowledge and routines for renal follow-up – a questionnaire study

**DOI:** 10.1186/s12882-018-0835-3

**Published:** 2018-02-13

**Authors:** I. Cockerell, M. Guenin, K. Heimdal, M. Bjørnvold, K. K. Selmer, O. Rouvière

**Affiliations:** 1 Department of Rare Disorders and Disabilities, Oslo University Hospital, National Centre for Rare Epilepsy-Related Disorders, Pb 4950, Nydalen, 0424 Oslo, Norway; 2 Department of Urinary and Vascular Imaging, Hôpital Edouard Herriot, Lyon, France; 3 Department of Medical Genetics, Oslo University Hospital, Oslo, Norway; 4 National Centre for Epilepsy, Division for Clinical Neuroscience, Oslo University Hospital, Oslo, Norway; 5 Department of Medical Genetics, Oslo University Hospital and University of Oslo, Oslo, Norway

**Keywords:** Tuberous sclerosis complex, Kidney, Angiomyolipoma, Follow up, Recommendations, Health literacy, Patient knowledge

## Abstract

**Background:**

Renal angiomyolipomas (AMLs) are a major clinical feature in patients with tuberous sclerosis complex (TSC). Spontaneous bleeding can be life threatening, and appropriate information and proper surveillance and management are important to limit morbidity and mortality. Because TSC is a rare disease, patients are at risk of suboptimal medical management. Our aim was to investigate patients’ and parents’ knowledge about renal angiomyolipomas (AMLs) in Tuberous Sclerosis Complex (TSC) and to identify current routines for renal follow-up.

**Methods:**

A questionnaire survey was initiated by the French Reference Centre on TSC. It was distributed in France through university hospitals and the patients’ association (2009-2011), and to patients registered by the Norwegian National Centre for Rare Epilepsy-Related Disorders (2013-2014). Contingency tables with Chi-Square test for independence (with Yates Continuity Correction) and Pearson-Chi-Square value were used for correlation statistics.

**Results:**

We included 357 patients (France, n=257; Norway n=100). Most participants knew that TSC is associated with AMLs. However, 42 % did not know about the risk of AMLrelated bleeding, and 37 % had been informed about the risk of bleeding only after the age of 15 years. Furthermore, 14 % did not know whether they themselves or their child had AMLs. Patients had less knowledge than parents. Medical consultations and patient associations were the main sources of information. Among 30 % of patients, renal imaging was not received at all, or not conducted every 1-3 years, as recommended by current guidelines. Regular imaging was more frequent in patients with AMLs < 15 years, than in patients with AMLs ≥ 15 years. Ultrasound was the most frequently used imaging modality.

**Conclusions:**

Knowledge of renal AML in TSC patients and their parents was lower than expected, and follow-up by renal imaging was suboptimal for a substantial proportion of patients. Patients and parents should be informed about the risk and symptoms of renal bleeding, at the latest when the patient is 15 years. Monitoring the growth of AMLs should be standardized to comply with guidelines. Transition between adolescence and adulthood is a high-risk period and ensuring appropriate follow-up at this time is particularly important.

## Background

Tuberous Sclerosis Complex (TSC) is a rare genetic disorder [1] characterised by benign tumours that can affect all organs (brain, kidneys, heart, lungs, and skin) at different stages in life [2, 3]. It is caused by mutations in the *TSC 1* gene on chromosome 9 or the *TSC 2* gene on chromosome 16 [1]. Epilepsy, neurocognitive impairments, autism, and dysfunctional renal and pulmonary organ systems [2, 3] are common symptoms.

Patients with TSC are at risk of suboptimal medical management for several reasons. First, it is a relatively rare disease affecting 1/6000 to 1/10000 live births [4–6]. Therefore, some physicians may not be very well conversant with the disease, and this may result in late diagnosis and inappropriate treatment. Second, as TSC is a multisystem disease affecting many organs, coordinated follow-up is necessary from different medical specialties including neurology, paediatrics, genetics, nephrology, urology, dermatology, pneumology, and imaging [2, 3, 7, 8]. Third, TSC is a chronic, lifelong disease, and thus requires a transition in care from the paediatric department to the adult department. During such a transition, there is a risk that important information might be mislaid and follow-up and management might suffer. Fourth, the clinical manifestations of TSC are highly variable [8], some mild forms of TSC may be found in adults with no particular history, and therefore may not be properly diagnosed or managed by general practitioners. Fifth, intellectual disability (ID) occurs in about half of TSC patients [9], and may be a barrier to diagnosis and optimal care [10].

These challenges make it important that TSC patients and their families receive correct and appropriate information on symptoms, with surveillance and access to potential treatments throughout life [7]. This is particularly true for renal manifestations of TSC that tend to increase during adolescence [11–13]. These manifestations vary considerably between patients [8] and are the leading cause of TSC-related deaths in adults [10, 14, 15]. TSC-associated renal manifestations include occurrence of renal angiomyolipomas (AMLs), cysts, and carcinomas, and occur in 48–85% of patients [10–13]. Spontaneous bleeding of an AML is one of the most serious renal complications [16], and, at worst, may be life-threatening, potentially necessitating emergency nephrectomy [10, 17]. Although the causes of AML bleeding are not well understood, the risk increases with AML size [16], and several guidelines recommend renal imaging every one to 3 years to monitor AML growth and to trigger prophylactic treatment for those AML considered to be at risk of bleeding [7, 13, 18].

To the best of our knowledge, studies investigating patients’ and parents’ knowledge of renal AMLs or describing the routine renal follow-up of TSC patients (including which imaging modality is used, at what frequency, and in which specific age groups) are lacking.

The French Reference Centre for TSC and the Norwegian National Centre for Rare Epilepsy-Related disorders have surveyed renal manifestations among TSC patients. The first part [19] investigated the prevalence of renal AMLs and AML-related bleedings among TSC patients. In the present paper we report the results of the second part, where the aim has been to investigate patients**’** and parents’ knowledge about renal AML in TSC and to identify current routines for renal follow-up.

## Methods

### Design of the study

In 2009, the French Reference Centre on TSC set up a national questionnaire survey on TSC renal manifestations in collaboration with the patient association. The French survey was distributed to TSC patients between November 2009 and June 2011 through specialist consultations at university hospitals (in paediatric, neurology, epilepsy, and nephrology departments). The questionnaire was sent by regular mail to all members of the French TSC patient association and distributed during their meetings. It was also available on their website.

Participants were asked whether they were aware that TSC was associated with AMLs and that AMLs could induce spontaneous haemorrhage. Participants were also asked whether the patients themselves had AMLs and how their renal follow-up was organized (age at first renal imaging, frequency, and imaging modality used for follow-up). The French questionnaire was anonymous, but patients were able to leave their names and contact details if they wished. In cases where responses required further clarification, a researcher (MG) contacted those patients who had left their contact details to obtain the necessary information.

The French questionnaire was adapted and translated into Norwegian and pilot tested in collaboration with the Norwegian patient association. From the registry of the Norwegian National Centre for Rare Epilepsy-Related Disorders, 214 were identified. Of these, 130 patients/parents who had consented to be contacted for research purposes were invited to participate by post between August 2013 and April 2014.

Those Norwegian patients/parents that did not answer the question about frequency and modality of renal imaging were sent reminders within 7 months of initial contact.

### Participants

A total of 357 participants completed questionnaires (257 in France, 100 in Norway). Of these questionnaires, 86 had been filled in by the patients themselves (24.1%), 256 by the patients’ parents (71.9%), and 13 by other persons (3.7%). In two cases (0.3%), the person who completed the questionnaire was not specified.

The median age of patients when the questionnaire was filled out was 26 years (min: 1 year-max: 72 years), 39% (139/354) were below 20 years and 56% (200/357) were females.

### Data analysis

Data from the questionnaires were coded and analysed with SPSS® (version 21). Contingency tables with Chi-Square test for independence (with Yates Continuity Correction); and Pearson-Chi-Square value were used for correlation statistics. A *p*-value of < 0.05 was considered statistically significant.

For all percentages presented in this article, the denominator corresponds to the number of participants who supplied an answer to the relevant question, and therefore may not correspond to the entire group of participants, as not all participants answered all the questions.

### Ethics

The study was approved by a Medical Ethics Committee, according to the national legislation in both countries.

The study was declared to the appropriate administrative authority (Commission Nationale de l’Informatique et des Libertés) in France and to the data protection officer in Oslo University Hospital in Norway.

In France, the questionnaire was anonymous and the study design was classified as ‘non-interventional’ by the Ethics Committee. As a result, the need for an informed consent was waived. Written informed consent to participate was received from participants in Norway from patients over 16 years of age and from parents or guardians of patients below 16 years and of patients who lacked competence to give consent.

## Results

### Participants’ knowledge about renal angiomyolipomas

In both France and Norway, the main sources of information about AMLs and the risk of renal bleeding were medical consultations and patient associations (Table 1).

**Table 1:** Patients’ sources of information on AML and the risk of renal bleeding

		Medical consultation n (%)	Patient association n (%)	Occurrence of renal symptoms n (%)	Internet n (%)	Medical journals n (%)
AML	Norway (*N* = 89)	71 (80)	37 (42)	14 (16)	18 (20)	12 (14)
	France (*N* = 242)	180 (74)	87 (36)	18 (7)	6 (3)	0
	Total (*N* = 331)	251 (76)	124 (37)	32 (10)	24 (7)	12 (4)
Risk of renal bleeding	Norway (*N* = 37)	26 (70)	17 (46)	1 (3)	3 (8)	6 (16)
	France (*N* = 162)	83 (51)	80 (49)	8 (5)	7 (4)	0
	Total (*N* = 199)	109 (55)	97 (49)	9 (5)	10 (5)	6 (3)

AML: angiomyolipoma

Approximately 7% of participants (23/356) stated that they were not aware that TSC was associated with AMLs. Patients completing the questionnaire themselves and patients in whom renal imaging was not performed at least every 3 years were significantly more likely to respond that they were not aware of this association (Table 2). In addition, 42% of participants (147/350) claimed to be unaware that AMLs carried a risk of bleeding. This percentage was significantly higher in Norway than in France, in patients who completed the questionnaire themselves, in patients in whom renal imaging was not performed at least every 3 years, and when the patients were < 15 years of age (Table 2).

**Table 2:** Awareness of AML and risk of bleeding among participants

		Number (%)	P
Participants unaware of AML association with TSC			
Country	Norway (*n* = 99)	9 (9)	0.21
	France (*n* = 257)	14 (5)	
Person who completed the questionnaire	Parent (*n* = 255)	8 (3)	< 0.001
	Patient (*n* = 86)	14 (16)	
Frequency of imaging	No or less than every 3 years (*n* = 83)	12 (16)	< 0.001
	At least every 3 years (*n* = 192)	6 (3)	
Patient’s age	< 15 years (*n* = 94)	4 (4)	0,355
	≥ 15 years (*n* = 259)	18 (7)	
Presence of AML	Yes (*n* = 189)	4 (2)	< 0.001
	No (*n* = 110)	3 (3)	
	Do not know (*n* = 50)	12 (24)	
Participants unaware of the risk of bleeding of AML			
Country	Norway (*n* = 98)	58 (59)	< 0.001
	France (*n* = 252)	89 (35)	
Person who completed the questionnaire	Parent (*n* = 250)	94 (38)	0.022
	Patient (*n* = 85)	44 (52)	
Frequency of imaging	No or less than every 3 years (*n* = 81)	46 (57)	0.001
	At least every 3 years (*n* = 190)	65 (34)	
Patient’s age	< 15 years (n = 94)	52 (55)	0.003
	≥ 15 years (*n* = 253)	95 (38)	
Presence of AML	Yes (*n* = 185)	45 (24)	
	No (n = 110)	59 (54)	< 0.001
	Do not know (*n* = 49)	37 (76)	

Percentages are based on the number of participants who answered the relevant question

AML: angiomyolipoma; TSC: tuberous sclerosis complex

**Fig. 1:**
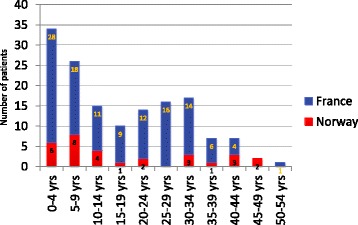
Histogram illustrating age at receiving information on risk of bleeding related to renal AML. Age at receiving information on risk of bleeding related to renal AML in the Norwegian population, red part of column. Age at receiving information on risk of bleeding related to renal AML in the French population, blue part of column

Among the participants who were aware about the risk of bleeding and who reported their age when receiving that information, 50% (75/149) received information before the age of 15 years, 27% (40/149) received information between 15 and 30 years, and 23% (34/149) did not receive information until after the age of 30 years (Fig 1). Among the 74 participants who were informed about bleeding risks when the patient was older than 15 years, 19 had been diagnosed with TSC after the age of 15 years. This means that 55/149 patients (37%) were diagnosed before they were 15 years old, but informed about the risk of bleeding complications after the age of 15 years. Among these 19 patients who had been diagnosed with TSC after the age of 15 years, 79% (15/19) had received information on the risk of AML bleeding within the first 3 years after their diagnosis, but four only received information 11–22 years after their diagnosis.

Approximately 14% of participants (50/350) did not know whether they had AMLs or not, and this group of participants were significantly more likely than the other participants to be unaware that TSC was associated with AMLs and also to be unaware of the bleeding risk of AMLs (Table 2).

### Renal monitoring in France and Norway

Imaging at least every 3 years had not been performed for 30% of the patients (83/276) and, among these, 24 had never undergone any renal imaging. Among those who had not undergone any imaging, seven were under 15 years, three were between 15 and 29 years, eight were between 30 and 44 years, and five were over 45 years. In one case, the patient’s age was not provided. The proportion of patients who did not receive imaging at least every 3 years was significantly higher among patients in France (36% [65/182]) than in Norway (19% [18/94]; *p* = 0.004).

In the group of patients with known AMLs, 19% (31/164) had not received imaging at least every 3 years (22% [26/121] in France versus 12% [5/43] in Norway; *p* = 0.15). Patients with AMLs who were younger than 15 years were more likely to receive regular imaging than patients with AMLs older than 15 years (93% [27/29] versus 77% [104/135]; *p* = 0.05).

Ultrasound was the most frequent imaging modality used in both countries. Only 33% of patients (56/169) reported receiving MRI or CT every 1–3 years, of whom six were under 20 years of age.

## Discussion

Our study showed that patients’ and parents’ knowledge about TSC renal AMLs was far from optimal. Although most participants knew that TSC is associated with AMLs, a considerable proportion (42%) was unaware that AMLs could bleed. More worryingly, 14% of the participants did not know if they had AMLs.

Knowledge of the natural history of AMLs is limited [20], but it is well known that the risk of bleeding increases when the diameter is > 4 cm [11, 21]. Presence of micro-aneurysms may also predispose to bleeding [21, 22]. Unfortunately, the presence of micro-aneurysms can only be assessed by intra-arterial angiography and this criterion cannot be used on a routine basis [23].

Lack of knowledge about the risk of bleeding was found to occur more frequently in the group of patients without AML (54%) than in patients with AML (24%, Table 2). Information provided to patients should be sufficient to ensure that they have adequate knowledge, but without causing unnecessary anxiety. This balance can be difficult to achieve.

Whether patients without AML or those considered to have a low risk of bleeding should be informed about this risk is debatable. In our opinion, those patients that do not have AML, or only small AMLs, should be informed that their risk is minimal; this provides information but should not provoke unnecessary worry.

AMLs usually appear during childhood [11–13]. The mean/median age of first detection of a renal abnormality (cyst or AML) in TSC ranges from 7.2 to 11.3 years [12, 24–27]. AMLs mostly grow during the second decade of life [25, 26] and bleeding complications are rare before 18 years. Previous analysis from the same group of patients reported an absence of bleeding before 16 years of age, but a marked peak in bleeding complications between the ages of 20 and 30 years, both in France and Norway [19]. It therefore could be argued that it is not essential for patients and their parents to be provided with information on renal manifestations of TSC during childhood. Some parents with young children with severe epilepsy may be overwhelmed by the descriptions of complications occurring in adolescence or adulthood. This may explain why more than 50% of patients below 15 years and their families were unaware of the risk of bleeding. Whereas some patients or their parents may want to know about all the risks related to the disease immediately, others might not want to receive all the information at once. All should be provided with information about potential renal complications of TSC by the risk age for bleeding (15 years), or earlier if they are considered to be at risk. Information should also be provided about the symptoms of renal bleeding: flank pain, haematuria, and shock [13, 16, 20], and patients and their care givers should know what they should do if these symptoms occur. In the group of patients ≥15 years, a substantial proportion did not know about the bleeding risk of AMLs. Even when the patients who were diagnosed with TSC after the age of 15 years are excluded, a substantial proportion 55/149 (37%) received this information inappropriately late. The medical community needs to improve their dissemination of information to patients and their families, and to adapt this information to patients’ ages and individual risk factors for bleeding.

That knowledge about AMLs and related bleeding complications was lower in patients than in parents may be associated with patients’ learning difficulties or ID. It should be noted that there is a risk that the symptoms of these patients may be underestimated by their caregivers [28], with assessment of symptomatic AMLs described as being almost impossible in some patients due toID. In some cases, only severe behavioural changes have alerted their carers to their patients’ condition [29]. In addition, a substantial proportion of adults with ID lack someone to talk to about their health; they may ignore symptoms and not tell anyone when they are in pain [28].

The important role of patient s’ associations as sources of information is demonstrated by these being one of the main sources of information in France and Norway. Although the Internet is widely recognised as being a rapidly expanding source of healthcare information [30], surprisingly few reported it as a source of information. However, the importance of the Internet as an information source is probably under-reported in our study. First, although the questionnaire did not ask for specification, the patient associations’ websites probably played an important role in providing information. Second, the Internet was not formally mentioned in the French survey. In addition, as much of the information available on the Internet may be only in English, language difficulties may also limit its use among people who are not confident in reading English. The National Centre for Rare Epilepsy-Related Disorders in Norway has published an online TSC guide, written in Norwegian [31], with management and surveillance recommendations for physicians, healthcare professionals, caregivers, and patients. The intention is to increase knowledge of TSC, including renal AMLs and their associated risk of bleeding amongst these groups in Norway.

Imaging follow-up was inadequate in many of the patients in our study. A substantial proportion of patients (36% in France, 19% in Norway) did not receive renal imaging at least every 3 years, although regular renal imaging is recommended [7, 13]. In addition, although the risk of developing symptomatic AML increases with age [10, 19] and increased monitoring is recommended in adulthood [7, 26], the percentage of patients undergoing regular imaging was significantly higher in patients under 15 years. The reasons for increased monitoring in these younger patients are unclear, but may reflect increased awareness of TSC renal manifestations among neuro-paediatricians and paediatricians than among physicians for adults. Surveillance and treatment for TSC patients are not centralized in France and Norway, which probably could have facilitated regular imaging. ID and communication problems in adult patients with TSC may also be a contributory reason [10]. Patients with mild forms of TSC may also be at risk of inappropriate follow up because of late diagnosis and lack of contact with the health services. Ultrasound was the most frequent imaging modality used in our population. There is no clear consensus on the optimal imaging modality for follow-up [7, 13]. Ultrasound is relatively cheap and safe (based on non-ionizing radiation), but provides less reliable measurements of large AMLs, especially when they are coalescent. CT and MRI are more accurate, but more expensive and time consuming [32, 33]. Because of short acquisition times with CT, it may be possible to avoid general anaesthesia in patients with ID, but repeated CT exposes patients to high doses of radiation. MRI is a non-ionizing radiation technique, but its long acquisition times may mean that general anaesthesia is necessary for some patients [32]. A combined MRI (or CT) of abdomen and brain for patients in need of both, may reduce stress and facilitate regular imaging. The most recent recommendations favour CT or MRI, at least in adults [7, 13]. The more frequent use of ultrasound in our study may reflect older recommendations [18, 25, 26, 34].

Although similar trends were found in France and Norway, there were some differences between the two countries. Patient information was significantly better in France, but regular imaging follow-up was performed in significantly more patients in Norway. These disparities may reflect differences in healthcare organization. Although the follow-up guidelines were probably not implemented in 2013–2014, when the study was conducted in Norway, awareness regarding kidney changes and the need for regular investigations may have been greater at that time than in 2009–2011 when the study was conducted in France [7, 13].

Our study has some limitations. First, it is limited by selection bias. The response rate was only known in Norway and was 47% (100/214).The survey mostly targeted patients known from specialized hospital institutions or patient associations. These patients probably have the best information and follow-up, and therefore, in the wider patient group, information and follow-up may be poorer than our results indicate. Second, our results were mostly based on patients’ or caregivers’ responses; these may be inaccurate, particularly concerning imaging follow-up. Third, a substantial proportion of patients in both countries did not answer the questions regarding imaging follow-up. Reminders were sent to non-responders in Norway, resulting in a higher response rate in Norway (94%) than in France (68%).

## Conclusion

Our study showed that patient information on renal AMLs in TSC and renal imaging follow-up were suboptimal in a substantial proportion of participants in Norway and France. Because renal bleeding complications usually occur after the age of 15 years, physicians and specialist nurses should ensure that patients, care givers and their families are properly informed on the risk of renal bleeding, at the latest when the patient is 15 years old. In addition, it is important that provision of advice is based upon the extent of renal manifestations in the individual patients. Monitoring of AML growth in both countries should be standardized in order to comply with current guidelines. The high-risk period between adolescence and adulthood should be of particular concern, and efforts should be directed towards improving collaboration between paediatricians and physicians for adults and to ensure appropriate follow-up in patients with ID and patients with mild forms of TSC.
